# Can N-Doped Biochar Achieve Safe Vegetable Production in Soil Heavily Contaminated by Heavy Metals?

**DOI:** 10.3390/toxics13020079

**Published:** 2025-01-23

**Authors:** Ming Chen, Yangzhou Wang, Junchao Pan, Lin Zhong, Mengjiao Qiao, Chenyang Gao, Tianqi Li, Yangyang Wang

**Affiliations:** 1 National Demonstration Center for Environmental and Planning, College of Geography and Environmental Science, Henan University, Kaifeng 475004, China; chenming@henu.edu.cn (M.C.); wangyangzhou@henu.edu.cn (Y.W.); panjunchao@henu.edu.cn (J.P.); zhonglin9@henu.edu.cn (L.Z.); qiaomengjiao@henu.edu.cn (M.Q.); gaochenyang1113@163.com (C.G.); 2 Key Laboratory of Geospatial Technology for the Middle and Lower Yellow River Regions, Henan University, Ministry of Education, Kaifeng 475004, China; 3 Henan Engineering Research Center for Control & Remediation of Soil Heavy Metal Pollution, Henan University, Kaifeng 475004, China

**Keywords:** health risk assessment, modified biochar, remediation, spinach, stabilization

## Abstract

Although the cultivation of food crops in farmland heavily contaminated by heavy metals is prohibited in China, vegetables can still be planted on a small-scale due to their short growth cycles and flexible sale models, posing a significant threat to local consumers. In this study, a pot culture experiment was conducted to investigate the feasibility of safe production through the in-situ stabilization of heavy metals in heavily contaminated soil. The remediation efficiency of wheat straw biochar and N-doped biochar, the growth of spinach, the heavy metal accumulation in spinach, and potential health risks were also explored. The results indicated that both biochar and N-doped biochar significantly affected the soil pH, cation exchange capacity, organic matter, available phosphorus, available potassium, alkaline nitrogen content, and spinach biomass, but the trends were variable. Additionally, the diethylenetriaminepentaacetic-extractable Pb, Cd, Cu, Zn, and Ni concentrations decreased 9.23%, 7.54%, 5.95, 7.44%, and 16.33% with biochar, and 10.46%, 12.91%, 21.98%, 12.62%, and 12.24% with N-doped biochar, respectively. Furthermore, N-doped biochar significantly reduced the accumulation of Pb, Cd, and Ni in spinach by 35.50%, 33.25%, and 30.31%, respectively. Health risk assessment revealed that the non-carcinogenic risk index for adults and children decreased from 17.0 and 54.8 to 16.3 and 52.5 with biochar and 11.8 and 38.2 with N-doped biochar, respectively, but remained significantly higher than the acceptable range (1.0). The carcinogenic risk assessment revealed that the risk posed by Cd in spinach exceeded the acceptable value (10^−4^) for both adults and children across all treatments. These results may imply that biochar and N-doped biochar cannot achieve the safe production of vegetables in soil heavily contaminated by heavy metals through in-situ stabilization.

## 1. Introduction

A large number of heavy metals (HMs) have been released into the environment due to rapid industrialization and urbanization in recent decades [1,2]. As the ultimate reservoir of various contaminants, soil accumulates most of these HMs through multiple pathways (atmosphere deposition, sewage irrigation, and the application of chemical fertilizers), resulting in the severe contamination of agricultural soils [3,4]. Previous studies have reported that more than half of the world’s soil is either slightly or severely contaminated with HMs, especially in developing countries [5,6,7]. Furthermore, HMs are toxic, non-biodegradable, and persistent, and can also accumulate through the food chain, posing significant threats to human health and the stability of soil ecosystems [8,9].

As a rapidly developing country, the quality of soil in China has seriously deteriorated [10]. The National Soil Pollution Investigation Bulletin (2014) of China reported that more than 10% of agricultural soils in China are contaminated with HMs [11]. However, the limited land resources and large population (over 1.4 billion in 2023) in China mean that most of these HMs contaminated agricultural soils must still be cultivated [12]. As a result, the HM content in agricultural products (includes wheat, rice, vegetables, and fruits) in some regions of China has exceeded the national limits, posing significant health risks to local residents [13,14]. Therefore, the safe and appropriate utilization of HMs contaminated farmland is of critical importance for local residents.

In-situ stabilization using amendments is considered to be a viable method for the safe utilization of HMs contaminated farmland [15]. Various materials have been used as amendments in previous studies, such as palygorskite, naosilica, and biochar [16,17,18]. Among them, biochar stands out prominently and has attracted great attention due to its environmentally friendly properties and large surface area [19,20,21]. Previous studies have demonstrated that biochar and modified biochar can reduce the accumulation of HMs in the edible parts of crops, as well as improve soil fertility, enhance microbial activity, and promote crop growth [22,23,24]. For example, Shen et al. [25] reported that MgO-coated corncob biochar can effectively reduce Pb leaching by 50.71% compared with unmodified biochar; Egene et al. [26] synthesized a holm oak biochar by pyrolyzing at 650 °C, and it exhibited a 66% and 77% reduction in Cd and Zn concentrations in soils, respectively; Huang et al. [27] constructed a bacteria-loaded biochar composite, which can significantly decreased the content of As and Cd to 0.34 and 0.075 mg/kg, respectively, in water spinach. However, most of these studies focused on the safe utilization of slightly or moderately contaminated farmland, while the potential for biochar and modified biochar to enable safe crop production in heavily HMs contaminated farmland remains unclear.

In fact, the cultivation of food crops in farmland heavily contaminated by HMs is prohibited in China, and most of these areas have been repurposed for the planting of trees, flowers, or energy crops [28]. However, vegetables can still be cultivated on a small scale due to their short growth cycles (even planting under trees) and flexible sale methods (local market or street vendors), which increases the supervisory difficulty of the local government [29]. Consequently, there are still some vegetables with a high HMs content being sold and consumed in region heavily contaminated by HMs, posing significant health risks to local consumers. Therefore, investigating the feasibility of using biochar and modified biochar to achieve the safe production of vegetables in a region heavily contaminated by HMs is of great practical significance.

Spinach, a fast-growing leafy vegetable with a high biomass, is commonly grown in the northern regions of the Qinling Huaihe River [30]. Spinach contains virous nutrients, including carbohydrates, vitamins, dietary fiber, and trace elements, which make it popular in daily diets [31]. However, spinach possesses a strong capacity to accumulate HMs, which significantly increased its health risk in food chains [32]. Therefore, the exploration of HMs in spinach is critical for lowering the health risk to local vegetable consumers. The aims of the present study were to evaluate: (a) the effects of N-doped biochar (HNC) on the properties of soil heavily contaminated by HMs and the growth of spinach; (b) the effects of HNC on the bioavailability of HMs and their accumulation in spinach; (c) the effects of HNC on the potential health risksthrough the consumption of these spinaches. The results of this study can provide reasonable suggestions for safe utilization of farmland heavily contaminated by HMs.

## 2. Materials and Methods

### 2.1. Soil Sample, Biochar, and N-Doped Biochar

The soil samples were collected from the farmland (0 to 20 cm depth) near a Pb/Zn smelter in Jiyuan, Henan Province, China (112°33′2.9″ E, 35°8′27.8″ N). According to the WRB classification [33], the soil was identified as *Calcaric Leptic Luvisol* (*Loamic*, *Aric*, *Ochric*). The collected soil samples were first air-dried and ground to <1 mm, then mixed thoroughly for homogenization for the pot experiment. In total, 10 g of ground soil (<1 mm) was further ground and passed through a 0.15 mm sieve for analysis of the soil’s properties. The selected properties of the soil are shown in Table 1.

The biochar (BC) and N-doped biochar (HNC) were prepared using wheat straw, as described in our previous study [36]. Briefly, the wheat straw was washed alternately with tap and deionized water, then cut into approximately 2 cm pieces and pyrolyzed under N_2_ protection at 350 °C for 2 h. The pyrolyzed product was ground to pass through a 100-mesh sieve and named BC. Furthermore, HNC was prepared by successively modified BC with KOH (2 mol/L), HNO_3_ (1 mol/L), and ammonia solution (5%, *w*/*w*). The pH of the BC and HNC was 8.60 and 5.51, respectively. HNC can effectively absorb various HMs in an aqueous solution, with the maximum adsorption capacities of 18.36 mg/g for Cu^2+^, 22.83 mg/g for Cd^2+^, and 49.38 mg/g for Pb^2+^, respectively. The physiochemical properties of BC and HNC are shown in Table S1.

### 2.2. Experimental Design

The pot culture experiment was conducted with seven treatments and four replications: 0% (CK), BC application (1%, 2%, and 3%, *w*/*w*), and HNC application (1%, 2%, and 3%, *w*/*w*). The basic fertilizer had been properly added before the seeds were sown. Briefly, 0.15 g of nitrogen (urea) and 0.18 g of phosphorus and potassium (potassium dihydrogen phosphate) were applied for per kg of soil, respectively. This applied content was not changed for BC or HNC. Each pot contained 1.0 kg of contaminated soil mixed thoroughly with a specific amount of BC or HNC. The water content was maintained at approximately 60% using deionized water, and then the soil was aged for 15 days before sowing the spinach seeds (10 seeds per pot, the variety of spinach seeds is “Savoy Spinach”, a typical spinach in North China fresh produce markets). After the seedlings reached a height of 5 cm, they were thinned to five plants per pot. The pots were repositioned irregularly every three days.

After seven weeks, the spinach plants were harvested and washed sequentially with tap water and deionized water to remove any adsorbed particulate matter. The plants were separated into aboveground and underground parts, and the fresh weight of both parts was recorded after they had dried naturally. The plant samples were placed in a baking oven at 80 °C to determine the dry weight and HM contents. At the same time, the soil samples were divided into two parts: one part was air-dried and sieved through 1 mm and 0.15 mm sieves for HM (DTPA-extractable) and soil property analyses (pH, available P (AP), available K (AK), alkaline N (AN), cation exchange capacity (CEC), and organic matter (SOM)).

### 2.3. Analytical Methods

The soil pH was measured using the potentiometric method according to the standards of HJ 962-2018 [37]. The AP and AK of these soil samples were determined using molybdenum antimony colorimetry and flame photometry method, respectively. The soil SOM was analyzed using the wet oxidation method according to the standards of NY/T 1121.6-2006 [38]. The CEC and total HM contents in the soil were determined based on our previous research [34]. The soil alkaline N was analyzed using the DB51T 1875-2014 [39]. The DTPA-extractable HMs were extracted using DTPA solution (GB/T 23739-2009) [40]. The details of the standard methods for analyzing the above parameters are presented in the Supplementary Materials (Text S1). The content of HMs (Pb, Cd, Cu, Zn, and Ni) in spinach tissues was analyzed using ICP-MS after being digested by HNO_3_-HClO_4_. All analyses of the soil and spinach tissues samples were performed in triplicate, and GBW07413 and GBW10011 were used as the standard materials for soil and plant, respectively. The recoveries of these HMs were between 91.5% and 107.4%.

### 2.4. Bioaccumulation Factor (BCF)

The bioaccumulation capacity of Pb, Cd, Cu, Zn, and Ni in the edible part of the spinach (fresh weight) was assessed using the BCF index:

(1) $$BCF=\frac{C_{plant}}{C_{soil}}$$

where *C_plant_* refers to the total HM content in the aboveground part of the spinach (mg·kg^−1^), and *C_soil_* refers to the total HM content in the soil (mg·kg^−1^).

### 2.5. Health Risk Assessment

The potential non-carcinogenic risk from the consumption of HM-containing spinach was evaluated by the target hazard quotient (HQ) and the hazard index (HI). The HQ and HI were calculated using the following equations:

(2) $$EDI=\frac{C_{i}\times IR\times ED\times EF}{BW\times AT\times 365}$$

(3) $$HQ=\frac{EDI}{RfD}$$

(4) $$HI=HQ_{1}+HQ_{2}+\ldots +HQ_{n}$$

where the EDI is the chronic daily intake of HMs through spinach ingestion (mg·kg^−1^·day^−1^); C_i_ is the HM content in the edible part of the spinach (mg·kg^−1^, fresh weight); IR is the daily intake of the spinach (kg·day^−1^); ED is the exposure duration (a); EF is the frequency of exposure (d·a^−1^); BW is the average body weight (kg); AT is the average exposure time (a); and RfD is the safe level of exposure to HMs over a lifetime (mg·kg^−1^·day^−1^). The detailed values of these parameters are listed in Table S2.

The lifetime probability of an individual developing cancer due to exposure to HMs through the consumption of spinach was evaluated using the target carcinogenic risk (TCR). The TCR value was calculated as per the following equation:

(5) TCR=EDI×SF

where SF is the slope factor of carcinogenic metals, the detailed values were selected as 6.1 for Cd, 0.0085 for Pb, and 0.84 for Ni according to the EPA-recommended HRA model [41,42].

### 2.6. Statistical Analysis

All data were processed using Microsoft Excel 2010, and the experimental results were expressed as the means ± standard deviations. The significance testing was conducted using one-way ANOVA with multiple comparisons (SPSS 25.0, Tukey, *p* < 0.05). Redundancy analysis (RDA) was performed using R (Version 3.5.0). Origin 2018 was used for graphing.

## 3. Results and Discussion

### 3.1. Effect of Biochar on Soil Properties

The changes in soil properties are presented in Table 2. The application of BC slightly increased the soil pH from 8.76 to 8.89 (*p* > 0.05). The pH of BC (8.60) is lower than that of the initial soil pH (8.76), indicating that the increase in the soil pH can be attributed to the gradual release of the alkaline minerals (metal hydroxides and carbonates) in BC [42]. In contrast, the application of HNC significantly decreased the soil pH from 8.76 to 8.26 (*p* < 0.05). The modification (oxidation by HNO_3_) removed the alkaline minerals from BC and decreased its pH to 5.51, which may be the primary reason for the reduction in soil pH. Several previous studies have emphasized the importance of the ‘liming effect’ for biochar in terms of its stabilizing of HMs in soil [43,44,45]. Therefore, the decrease in soil pH may adversely affect the stabilization of HMs.

The application of BC slightly decreased the soil AN (*p* < 0.05), which can be explained by the low N content in BC (0.51%) and the slight increase in soil pH (enhanced ammonia volatilization) (Table S1) [46]. The modification significantly increased the total N content from 0.51% (BC) to 4.44% (HNC), leading to a substantial rise in soil AN from 40.44 to 85.24 mg/kg after HNC application (*p* < 0.05). Furthermore, BC application significantly increased soil AP from 30.92 to 36.63 mg/kg (*p* < 0.05), whereas HNC only slightly increased the AP from 30.92 to 33.86 mg/kg (*p* > 0.05). The soil AK significantly increased from 262.10 to 829.84 mg/kg after the application of BC (*p* < 0.05), whereas the influence of HNC on soil AK was insignificant (*p* > 0.05), likely due to the high AK content of BC (6.19 g/kg, Table S1). There results indicate that the modification process (oxidation by HNO_3_) removed certain nutrient elements from BC and may have adverse effects on soil fertility and crop growth.

### 3.2. Biomass of Spinach

**Figure 1:**
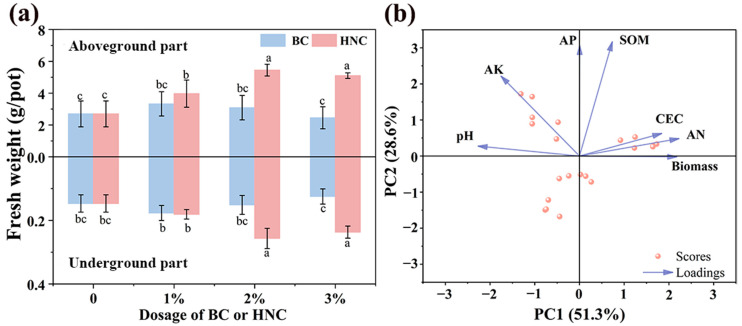
(**a**) Biomass of spinach (mean ± SE; n = 4) and (**b**) principal component analysis of correlation between soil properties and spinach biomass (different lowercase letters indicate significant differences among different treatments, *p* < 0.05).

The application of BC resulted in variable changes in both aboveground and underground spinach biomass, ranging from 2.70 to 3.33 g/pot and 0.15 to 0.18 g/pot, respectively (Figure 1a). However, these changes were insignificant (*p* > 0.05), except for the underground biomass at the 3% application rate. In contrast, HNC increased spinach biomass from 2.70 to 5.45 g/pot in the aboveground part and from 0.15 to 0.27 g/pot for the underground part. Spinach biomass (both aboveground and underground) significantly increased with the application rate of HNC increasing from 0% to 2% (*p* < 0.05). However, a further increase in HNC application rate (3%) slightly decreased spinach biomass (both aboveground and underground) compared to the 2% application rate, but this change was insignificant (*p* > 0.05). These results indicate that HNC is more beneficial for spinach growth compared with that of the BC and may reduce HM content in spinach through the ‘dilution effect’ [47].

Principal component analysis (PCA) showed that spinach biomass was positively correlated with AN, CEC, and SOM, and negatively correlated with the soil pH and AK (Figure 1b). In addition, HNC significantly increased the AN, CEC, SOM, and AP, and decreased the soil pH (Table 2), which likely explains the observed increase in spinach biomass.

### 3.3. Effect of BC and HNC on Bioavailable HMs in Soil

**Figure 2:**
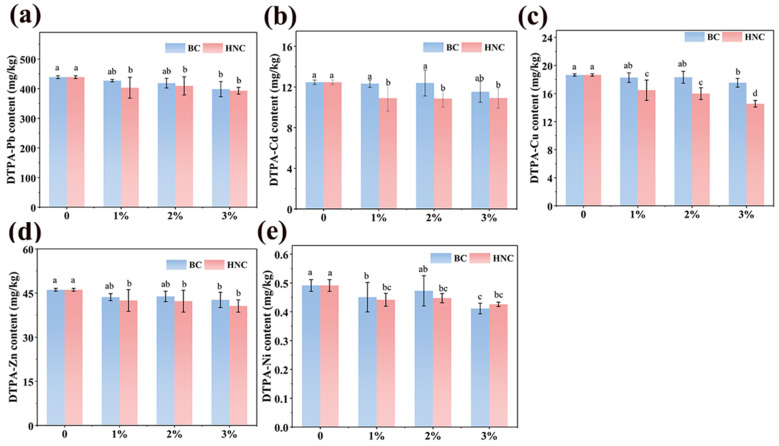
The contents of DTPA-extractable Pb (**a**), Cd (**b**), Cu (**c**), Zn (**d**), and Ni (**e**) in the soil (mean ± SE; n = 4) (different lowercase letters indicate significant differences among different treatments, *p* < 0.05).

The DTPA-extractable HMs in the heavily contaminated soil decreased slightly as the application rates of BC and HNC increased (Figure 2). Specifically, the DTPA-extractable Pb, Cd, Cu, Zn, and Ni decreased from 439.0, 12.47, 18.65, 46.12, and 0.49 mg/kg to 398.5, 11.53, 17.54, 42.69, and 0.41 mg/kg after the application of BC. Similarly, their concentrations decreased to 393.1, 10.86, 14.55, 40.60, and 0.43 mg/kg after the HNC was applied. The highest stabilization efficiency of Pb, Cd, Cu, Zn, and Ni were 9.23%, 7.54%, 5.95, 7.44%, and 16.33% with BC, and 10.46%, 12.91%, 21.98%, 12.62%, and 12.24% with HNC, respectively. The higher stabilization efficiency of HNC compared with that of the BC can be attributed to its higher content of oxygen-containing functional groups and enhanced adsorption capacity for HMs [36]. However, the stabilization efficiencies of HNC were still much lower than in many previous studies [45,47,48], which can be attributed to its higher initial HM concentrations in the soil.

Several modified biochars have been employed to stabilize HMs in soil with relatively high stabilization efficiencies. Phosphorus-loaded biochar reduced the bioavailable Cd in soil by approximately 65% with an application rate of 3% (*w*/*w*) [45]. Nanoscale zero-valent iron-supported biochar could simultaneously stabilize Cd and As in soil, with stabilization efficiencies of 34.93% and 32.64%, respectively [47]. Multiple modified biochar could reduce the DTPA-extractable Cd, Pb, and Cu in soil by 83.76%, 47.94%, and 100%, respectively [48]. However, most of the research focused on the stabilization of slightly or moderately HM-contaminated soil.

In the present study, the immobilization amount (of metals immobilized) of HNC is 1.53 mg/g for Pb, 0.053 mg/g for Cd, 0.137 mg/g for Cu, 0.184 mg/g for Zn, and 0.03 mg/g for Ni, respectively, which exhibit almost no difference relative to multiple modified biochar, nanoscale zero-valent iron-supported biochar, and other high-immobilization efficiency biochar materials (Table S3). However, the residual DTPA-extractable heavy metals in soil (439.0, 12.47, 18.65, 46.12, and 0.49 mg/kg for Pb, Cd, Cu, Zn, and Ni, respectively) are still much higher than the initial concentrations in many previous studies [49,50,51], which can partially explain the low HM stabilization efficiency of HNC. On the other hand, soil environmental factors (such as pH, cations contents, and micro-organism, etc.) can greatly influence biochar immobilization efficiency [52]. Our studies revealed that the soil pH significantly decreased after the application of HNC, which is not helpful in terms of HM immobilization. Previous studies confirmed that HMs mainly exist in a binding state (exchangeable and reducible) in alkaline soils [53,54]. However, the decreased pH in the present study may cause certain HMs to leach, which can also reduce the stabilization efficiency of HNC for HMs.

In fact, it is widely acknowledged that soil heavily contaminated by HMs is unsuitable for crop cultivation. Most of these heavily contaminated agricultural lands have been converted to forest or grassland. However, due to the limited farmland in China, vegetables can be grown on a small scale and with short growth cycles, which complicates local government oversight. Therefore, exploring the possibility of safe vegetable production in soils heavily contaminated by HMs is of great significance to local residents. Unfortunately, in the present study, the highest stabilization efficiencies of Pb, Cd, Cu, Zn, and Ni just reached 10.46%, 12.91%, 21.98%, 12.62%, and 16.33%. This result may imply that the application of the N-doped biochar cannot achieve the safe production of vegetables in soil heavily contaminated by HMs.

### 3.4. Effect of BC and HNC on Accumulation of HMs in Spinach

**Figure 3:**
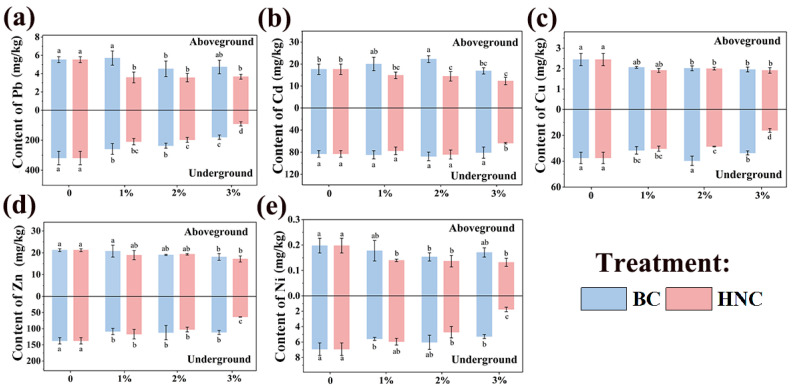
HM content of Pb (**a**), Cd (**b**), Cu (**c**), Zn (**d**), and Ni (**e**) in aboveground and underground part of spinach (mean ± SE; n = 4, different lowercase letters indicate significant differences among different treatments, *p* < 0.05).

The contents of Cu, Ni, and Zn in the aboveground part of spinach decreased with increasing application rates of BC, and the remediation efficiency of HNC was significantly higher than that of BC (Figure 3). Notably, the Ni concentration decreased most significantly after the application of 3% HNC, with a reduction rate of 33.25%. For Pb, the application of both BC and HNC significantly reduced its content in the aboveground part of spinach (except for 1% BC) (Figure 3a). The application of 2% HNC achieved the most significant reduction, with a decrease of 35.50%. With the increase in the BC application rates, Cd content in the aboveground part of spinach initially increased and then decreased, and the application of 1% and 2% BC resulted in a higher Cd content than the control (Figure 3b).

The Cd content gradually decreased with the increasing application rate of HNC, with the highest reduction rate of 30.31% observed after the application of 3% HNC. Furthermore, the content of Pb and Cd in the edible part of the spinach in all treatments exceeded the national standard of China (GB 2762-2017) (0.2 mg Cd/kg fresh weight, and 0.3 mg Pb/kg fresh weight, but Cu, Zn, and Ni content is not limited by the standard) [55]. These results further verified that modified biochar cannot realize the safe production of vegetables in soil heavily contaminated by HMs.

The bioaccumulation analysis indicated that the BCF of spinach for Cu, Zn, Cd, and Ni decreased to varying degrees after the application of BC and HNC (except for the 1% and 2% BC treatments), whereas their effect on Pb was most obvious. Spinach showed the lowest BCF for Cu, Zn, Cd, and Ni after the application of 3% HNC, with reductions of 21.88%, 19.04%, 30.32%, and 32.63%, respectively, compared to the control. Under the same treatment, the BCF of the five HMs ranked as: Cd > Zn > Cu > Ni > Pb, indicating that spinach had the highest accumulation ability for Cd and the lowest for Pb. Moreover, HNC was more effective in reducing the enrichment of Cu, Zn, Cd, and Ni in the edible parts of the spinach compared to BC (Table 3).

### 3.5. Health Risk Assessment

The non-carcinogenic risks of HMs through spinach consumption are shown in Table 4. The HI for both adults and children decreased from 17.0 to 16.3 and 54.8 to 52.5 after BC was applied, and from 17.0 to 11.8 and 54.8 to 38.2 after HNC was applied, respectively. Unfortunately, all HI values in the present study were significantly higher than the permissible limit (1.0) (Table S4), indicating that HMs in spinach still pose a serious non-carcinogenic risk to both adults and children. Notably, Cd accounted for more than 98% of the HI, while the non-carcinogenic risks posed by Cu, Zn, Pb, and Ni were within the acceptable range (Table 4). This result indicates that although Pb and Cd were all heavily contaminated in soil and spinach, Cd was the only element that posed a non-carcinogenic risk to spinach consumers.

The carcinogenic risk index (CR) for spinach consumption is shown in Table 5. The CR for Pb and Ni in spinach remains within the acceptable range for both adults and children. However, the CR of Cd exceeded the acceptable value (10^−4^) in all treatments (Table S4), indicating that Cd in spinach posed an unacceptable carcinogenic risk for both adults and children. Consequently, the TCR of Pb, Ni, and Cd via the consumption of spinach all exceeded the acceptable range for both adults and children, and Cd is the main contributor to carcinogenic health risks. Nevertheless, the CR of Pb, Ni, Cd, and the TCR all decreased obviously for both adults and children after biochar was applied, with the decrease after HNC being more significant than that of the BC.

## 4. Conclusions

In conclusion, both BC and HNC changed the properties of the soils, but this change was variable. The content of bioavailable heavy metals decreased gradually with the increase in both BC and HNC application rate. In addition, BC and HNC also reduced the accumulation of heavy metals in spinach, with their effect on Pb being the most obvious. Correspondingly, BC and HNC also reduced the hazard index and target carcinogenic risk posed to the local spinach consumers to some extent, but the potential risk is still unacceptable for both adults and children. The results of the present study may imply that in-situ stabilization cannot achieve the safe production of vegetables in soil heavily contaminated by heavy metals. A change in land use type may be the most practical method to reduce the potential risk of HMs in heavily contaminated regions.

## Figures and Tables

**Table 1:** Selected properties of the soil sample and related permissible values of HMs.

Soil Property	Value	Background Values [34]	Risk Screening Values [35]
pH	8.75 ± 0.15	-	-
Cation exchange capacity (cmol/kg)	18.28 ± 1.08	-	-
Organic matter content (g/kg)	31.15 ± 0.92	-	-
Total Pb (mg/kg)	851.0 ± 4.24	19.60	170
Total Cu (mg/kg)	95.50 ± 8.61	19.70	100
Total Cd (mg/kg)	20.00 ± 0.71	0.07	0.6
Total Zn (mg/kg)	242.0 ± 4.24	60.10	300
Total Ni (mg/kg)	20.75 ± 3.18	26.70	190

**Table 2:** Change in the soil physicochemical properties after the application of BC and HNC (The different letters in the same column mean a significant difference at *p* < 0.05).

Dosage	pH	SOM (g/kg)	CEC (cmol/kg)	AN (mg/kg)	AP (mg/kg)	AK (mg/kg)
CK	8.8 ± 0.2a	31 ± 1d	18 ± 1 c	40 ± 3a	31 ± 1c	262 ± 16de
1% BC	8.8 ± 0.1a	43 ± 2c	19 ± 1 c	40 ± 1a	30 ± 1c	489 ± 13c
2% BC	8.9 ± 0.1a	53 ± 3b	18 ± 2 c	37 ± 2ab	36 ± 1a	687 ± 11b
3% BC	8.8 ± 0.1a	61 ± 1a	18 ± 0c	35 ± 3b	37 ± 2a	830 ± 8a
1% HNC	8.5 ± 0.1b	44 ± 2c	20 ± 1b	59 ± 5c	33 ± 1b	258 ± 88d
2% HNC	8.4 ± 0.1bc	53 ± 3b	20 ± 0a	71 ± 2b	34 ± 1b	229 ± 30e
3% HNC	8.3 ± 0.1c	59 ± 1a	22 ± 1a	85 ± 3a	33 ± 1b	240 ± 10e

**Table 3:** The bioaccumulation factor (BCF) of spinach.

		BC				HNC		
		CK	1%	2%	3%	1%	2%	3%
BCF	Cu	0.0256	0.0216	0.0211	0.0205	0.0200	0.0209	0.0200
	Zn	0.0877	0.0857	0.0786	0.0748	0.0782	0.0799	0.0710
	Pb	0.0065	0.0067	0.0053	0.0056	0.0042	0.0042	0.0043
	Cd	0.8606	0.9778	1.0854	0.8252	0.7261	0.7041	0.5997
	Ni	0.0095	0.0086	0.0074	0.0082	0.0067	0.0066	0.0064

**Table 4:** Non-carcinogenic risk of HMs in spinach.

				BC			HNC		
			CK	1%	2%	3%	1%	2%	3%
HQ	Cu	Adult	5.8 × 10^−2^	4.9 × 10^−2^	4.8 × 10^−2^	4.6 × 10^−2^	4.5 × 10^−2^	4.7 × 10^−2^	4.5 × 10^−2^
		Children	1.9 × 10^−1^	1.6 × 10^−1^	1.5 × 10^−1^	1.5 × 10^−1^	1.5 × 10^−1^	1.5 × 10^−1^	1.5 × 10^−1^
	Zn	Adult	6.7 × 10^−2^	6.6 × 10^−2^	6.0 × 10^−2^	5.7 × 10^−2^	6.0 × 10^−2^	6.1 × 10^−2^	5.4 × 10^−2^
		Children	2.2 × 10^−1^	2.1 × 10^−1^	1.9 × 10^−1^	1.8 × 10^−1^	1.9 × 10^−1^	2.0 × 10^−1^	1.8 × 10^−1^
	Pb	Adult	1.5 × 10^−1^	1.5 × 10^−1^	1.2 × 10^−1^	1.3 × 10^−1^	9.7 × 10^−2^	9.6 × 10^−2^	9.9 × 10^−2^
		Children	4.8 × 10^−1^	5.0 × 10^−1^	4.0 × 10^−1^	4.1 × 10^−1^	3.1 × 10^−1^	3.1 × 10^−1^	3.2 × 10^−1^
	Cd	Adult	16.7	19.0	21.1	16.0	14.1	13.7	11.6
		Children	53.9	61.3	68.0	51.7	45.5	44.1	37.6
	Ni	Adult	9.4 × 10^−4^	8.4 × 10^−4^	7.2 × 10^−4^	8.1 × 10^−4^	6.6 × 10^−4^	6.5 × 10^−4^	6.3 × 10^−4^
		Children	3.0 × 10^−3^	2.7 × 10^−3^	2.3 × 10^−3^	2.6 × 10^−3^	2.1 × 10^−3^	2.1 × 10^−3^	2.0 × 10^−3^
HI		Adult	17.0	19.3	21.3	16.3	14.3	13.9	11.8
		Children	54.8	62.2	68.8	52.5	46.2	44.8	38.2

**Table 5:** The carcinogenic risk of spinach ingestion under BC and HNC treatment.

				BC			HNC		
			CK	1%	2%	3%	1%	2%	3%
CR	Pb	Adult	4.4 × 10^−5^	4.6 × 10^−5^	3.6 × 10^−5^	3.8 × 10^−5^	2.9 × 10^−5^	2.9 × 10^−5^	3.0 × 10^−5^
		Children	1.4 × 10^−4^	1.5 × 10^−4^	1.2 × 10^−4^	1.2 × 10^−4^	9.3 × 10^−5^	9.3 × 10^−5^	9.5 × 10^−5^
	Cd	Adult	1.0 × 10^−1^	1.2 × 10^−1^	1.3 × 10^−1^	9.8 × 10^−2^	8.6 × 10^−2^	8.3 × 10^−2^	7.1 × 10^−2^
		Children	3.3 × 10^−1^	3.7 × 10^−1^	4.2 × 10^−1^	3.2 × 10^−1^	2.8 × 10^−1^	2.7 × 10^−1^	2.3 × 10^−1^
	Ni	Adult	3.2 × 10^−4^	2.9 × 10^−4^	2.5 × 10^−4^	2.7 × 10^−4^	2.2 × 10^−4^	2.2 × 10^−4^	2.1 × 10^−4^
		Children	1.0 × 10^−3^	9.2 × 10^−4^	8.0 × 10^−4^	8.9 × 10^−4^	7.3 × 10^−4^	7.1 × 10^−4^	6.9 × 10^−4^
TCR		Adult	1.0 × 10^−1^	1.2 × 10^−1^	1.3 × 10^−1^	9.8 × 10^−2^	8.6 × 10^−2^	8.4 × 10^−2^	7.1 × 10^−2^
		Children	3.3 × 10^−1^	3.7 × 10^−1^	4.2 × 10^−1^	3.2 × 10^−1^	2.8 × 10^−1^	2.7 × 10^−1^	2.3 × 10^−1^

## Data Availability

The data that support the findings of this study are available from the corresponding author upon reasonable request.

## Supplementary Materials

The following are available online at https://www.mdpi.com/article/10.3390/toxics13020079/s1, Text S1: The detailed analysis procedure for soil parameters; Table S1: Physiochemical properties of BC and HNC; Table S2: The selection of the detailed values of these parameters; Table S3: The immobilization amount between HNC and other biochar materials; Table S4: The level of HQ, HI, and CR [3,34,45,48,49,50,51,56,57].
