# Supplementary material for: Can N-Doped Biochar Achieve Safe Vegetable Production in Soil Heavily Contaminated by Heavy Metals?
Source: Toxics. 2025 Jan 23;13(2):79. doi: 10.3390/toxics13020079 (PMC11860317; doi:10.3390/toxics13020079)
Supplement: Supplementary file 1 [file toxics-13-00079-s001.zip › toxics-3347139-supplementary.pdf]

Can N-Doped Biochar Achieve Safe Vegetable Production in Soil Heavily Contaminated by Heavy Metals?

Ming Chen[1], Yangzhou Wang[1], Junchao Pan[1], Lin Zhong[1], Mengjiao Qiao[1], Chenyang Gao[1], Tianqi Li[1,2*], Yangyang Wang[1,2,3**]

[1]National Demonstration Center for Environmental and Planning, College of Geography and Environmental Science, Henan University, Kaifeng 475004, China

[2]Key Laboratory of Geospatial Technology for the Middle and Lower Yellow River Regions (Henan University), Ministry of Education, Kaifeng 475004, China

[3]Henan Engineering Research Center for Control & Remediation of Soil Heavy Metal Pollution, Henan University, Kaifeng 475004, China

---

[*]Tianqi Li, Henan University, Kaifeng 475004, China; Tel.: +86-15203780115; Fax:+86-371-23881850; E-mail address: tianqi_li@henu.edu.cn

[**]Yangyang Wang, Henan University, Kaifeng 475004, China; Tel.: +86-15194602157; Fax:+86-371-23881850; E-mail address: wangyangyangxyz@163.com

Text S1 The detailed analysis procedure for soil parameters

1. pH

10 g of soil (1-mm) was added to a 50 mL beaker containing 25 mL CO_2 removed water, then mixing thoroughly with a glass rod for 1 min, and stand for 30 min before measuring with a calibrated pH mater.

2. Cation exchange capacity (CEC)

6.0 g of soil (1-mm) was added to a 100 mL centrifuge tube containing 33 mL CH_3COONa solution (1.0 mol/L, pH = 8.2), shake at 250 rpm on a rotary shaker for 5 minutes, then centrifuged for 5 min at 4000 rpm to remove the supernatant. The soil was re-suspended with 33 mL CH_3COONa solution (1.0 mol/L, pH = 8.2), then shaken at 250 rpm on a rotary shaker for 5 minutes, then centrifuged for 5 min at 4000 rpm to remove the supernatant. Repeat this step 4 times.

The soil was re-suspended with 33 mL absolute alcohol, shaking at 250 rpm for 5 min and centrifuging at 4000 rpm for 5 min to remove the supernatant. Repeat this step for 3 times.

After that, the soil was re-suspended with 33mL of CH_3COONH_4 (1 mol/L, pH = 7.0), shaking at 250 rpm for 5 min and centrifuging at 4000 rpm for 5 min, the supernatant was transferred into a 100 mL volumetric flask. Repeat this step 3 times. Then dilute the solution to 100 mL with CH_3COONH_4 solution. The content of Na^+ in the filtrate was determined by flame photometry after filtering through filter paper.

3. Organic matter content (SOM)

0.25 g of soil (0.15-mm) was added to thermostability test tube (30*200 mm), then 5 mL of $K_2Cr_2O_7$ solution (0.133 mol/L) and 5 mL H_2SO_4 (ρ = 1.84) were added. Then place the test tube

into a preheated oil bath (185 ºC), and control the temperature of the oil bath between 170 to 180 ºC for 5 min (start timing from bubbles appear inside the test tube). After that, take out the test tube and cool it to room temperature. Transfer the solution from the test tube into a 150 mL Erlenmeyer flask by rinsing with distilled water. 4 drops of phenanthroline indicator were added and shaken to evenly. Finally, the solution was titrated with calibrated $FeSO_4 \cdot 7H_2O$ (0.2 mol/L) solution until the color changes to red at the endpoint.

4. Available potassium (AK)

5 g of soil (1-mm) was added to a 150 mL Erlenmeyer flask containing 50 mL NH_4OAc solution (1 mol/L, pH = 7.0). The suspension was shaken evenly on a rotary shaker at 175 rpm for 30 min at 25 ºC, then filtered through filter paper. The content of K in the filtrate was determined using flame photometry.

5. Available phosphorus (AP)

2.5 g of soil (1-mm) was added to a 150 mL Erlenmeyer flask containing 50 mL $NaHCO_3$ solution (0.5 mol/L, pH = 8.5). The suspension was shaken evenly on a rotary shaker at 175 rpm for 30 min at 25 ºC, then filtered through phosphorus-free filter paper. 10 mL of the filtrate was transferred into a 50 mL colorimetric tube, and 10~20 mL (depended on the concentration of AP) distilled water was added, then 5 mL of molybdate was added by slowly dropping. Diluted with distilled water to 50 mL and mixed thorough. Then, the content of P was analyzed using ultraviolet and visible spectrophotometer at a wavelength of 700 nm after standing for 30 min.

6. Available nitrogen (AN)

2.0 g of soil (1-mm) was added to outer chamber of a diffusion boat, then 0.2 g of $FeSO_4 \cdot 7H_2O$ was added and mixed thoroughly. The diffusion boat was rotated to ensure even

distribution of the sample. Then, 2 mL of borate indicator solution (20 g/L, pH = 4.5) was added to the inner chamber of the diffusion boat. The alkaline gel was smeared on the exterior edge of the diffusion boat, a glass cover was placed on top and ensuring a complete seal between the diffusion boat and the glass. Open a crack between the glass cover and the diffusion boat, 10 mL of NaOH solution (1.0 mol/L) was added to the outer chamber through the crack and the diffusion boat was promptly sealed immediately, then incubated at 40 ºC for 24 h in a constant-temperature incubator. After that, the resulting solution (borate indicator solution) was titrated using a standard H_2SO_4 solution (0.005 mol/L).

7. DTPA- extractable HMs

5 g of soil (1-mm) was added to a 100 mL Erlenmeyer flask containing 25 mL DTPA solution (0.005 mol/L DTPA, 0.01 mol/L $CaCl_2$, 0.1 mol/L TEA, and pH=7.3). The suspension was shaken evenly on a rotary shaker at 175 rpm for 2 hours at 25 ºC, then filtered through filter paper. The content of HMs in the filtrate was detected by inductively coupled plasma-mass spectrometry (ICP–MS, ICAP 6200, USA Thermo Fisher).

Table S1 Physiochemical properties of BC and HNC

	BC	HNC
pH_{pzc}	4.77	2.69
carboxyl group (mmol/g)	1.251	2.320
lactonic group (mmol/g)	1.206	1.336
hydroxyl group (mmol/g)	1.975	1.670
C [%]	76.29	57.55
H [%]	3.88	6.26
O [%]	18.38	27.52
N [%]	0.51	4.44
H/C	0.05	0.11
O/C	0.24	0.48
(O+N)/C	0.25	0.56
BET surface area (m^2/g)	3.1764	2.7033
BJH pore volume (cm^3/g)	0.012958	0.010247
Average diameter of pores (nm)	10.198	13.464
Micropore volume (cm^3/g)	0.0007243	0.0004308
Available K (g/kg)	6.19	0.61

Table S2 The selection of the detailed values of these parameters

Parameters		Value
IR ($kg \cdot day^{-1}$)	Adult	0.244
	Children	0.186
ED (a)	Adult	70

	Children	12
EF (d·a^{-1})		365
BW (kg)	Adult	63.5
	Children	15
AT (a)		365×ED
R*f*D (mg·kg^{-1}·day^{-1})	Pb	0.0035
	Cd	0.001
	Cu	0.04
	Zn	0.3
	Ni	0.02

Note: These values were referenced from the report of Ruan et al. (2023) [1] and Liu et al. (2021) [2].

Table S3 The immobilization amount between HNC and other biochar materials.

Materials	Immobilization amount (mg/g)					Reference
	Pb	Cd	Cu	Zn	Ni	
Phosphorus-loaded biochar	0.56	0.25	0.50	1.17	/	[3]
Multiple-modified biochar	/	0.07	0.19	/	/	[4]
Mn-modified bamboo biochar	0.49	0.03	0.14	increased	/	[5]
Silica–magnesium coupling in lignin–based biochar	0.24	0.23	0.03	2.54	/	[6]
$MgCl_2$-modified lignin biochar	increased	0.07	0.03	4.11	/	[7]
corn stalk biochar	1.01	0.02	0.21	0.75	/	[8]
Ca-modified cypress biochar	/	0.30	0.02	0.08	/	[9]
HNC	1.53	0.05	0.13	0.18	0.01	This work

Table S4 The level of HQ, HI, and CR

HQ		HI		CR	
HQ≤1	No obvious noncarcinogenic risks	HI≤1	No obvious health risks	CR＜10^{-6}	No carcinogenic risk
HQ>1	Having adverse effects on human health	1<HI≤10	posed noncarcinogenic risk	10^{-6}≤CR≤10^{-4}	Acceptable carcinogenic risk
		HI>10	Heavily chronic toxicity	CR>10^{-4}	Unacceptable carcinogenic risk

References

1. Ruan, X.; Ge, S.; Jiao, Z.; Zhan, W.; Wang, Y. Bioaccumulation and risk assessment of potential toxic elements in the soil-vegetable system as influenced by historical wastewater irrigation. *Agric. Water Manag.* **2023**, *279*, 108197.
2. Liu, X.; Gu, S.; Yang, S.; Deng, J.; Xu, J. Heavy metals in soil-vegetable system around E-waste site and the health risk assessment. *Sci. Total Environ.* **2021**, *779*, 146438.
3. Ahmad, M.; Usman, A.; Al-Faraj, A.; Ahmad, M.; Sallam, A.; Al-Wabel, M. Phosphorus-loaded biochar changes soil heavy metals availability and uptake potential of maize (*Zea mays* L.) plants. *Chemosphere* **2018**, *194*, 327–339.
4. Wang, Y.; Zheng, K.; Zhan, W.; Huang, L.; Liu, Y.; Li, T.; Yang, Z.; Liao, Q.; Chen, R.; Zhang, C.; et al. Highly effective stabilization of Cd and Cu in two different soils and improvement of soil properties by multiple-modified biochar. *Ecotoxicol. Environ. Saf.* **2021**, *207*, 111294.
5. Su, J.; Guo, Z.; Zhang, M.; Xie, Y.; Shi, R.; Huang, X.; Tuo, Y.; He, X.; Xiang, P. Mn-modified bamboo biochar improves soil quality and immobilizes heavy metals in contaminated soils. *Environ. Technol. Innov.* **2024**, *34*, 103630.
6. Li, X.; Zhang, Y.; Huang, W.; Luo, Y.; Wang, J.; She, D. Silica–magnesium coupling in lignin–based biochar: A promising remediation for composite heavy metal pollution in environment. *J. Environ. Manag.* **2024**, *363*, 121392.
7. Li, X.; Yu, Y.; Zhang, Y.; Wang, J.; She, D. Synergistic effects of modified biochar and selenium on reducing heavy metal uptake and improving pakchoi growth in Cd, Pb, Cu, and Zn–contaminated soil. *J. Environ. Chem. Eng.* **2024**, *12*, 113170.
8. Huang, C.; Wang, W.; Yue, S.; Adeel, M.; Qiao, Y. Role of biochar and Eisenia fetida on metal bioavailability and biochar effects on earthworm fitness. *Environ. Pollut.* **2020**, *263*, 114586.
9. Wang, Y.; You, L.; Lyu, H.; Liu, Y.; He, L.; Hu, Y.; Luo, F.; Yang, S. Role of biochar–mineral composite amendment on the immobilization of heavy metals for Brassica chinensis from naturally contaminated soil. *Environ. Technol. Innov.* **2022**, *28*, 102622.
